# Inability of NS1 protein from an H5N1 influenza virus to activate PI3K/Akt signaling pathway correlates to the enhanced virus replication upon PI3K inhibition

**DOI:** 10.1186/1297-9716-43-36

**Published:** 2012-04-24

**Authors:** Weizhong Li, Gefei Wang, Heng Zhang, Yanqin Shen, Jianping Dai, Liqi Wu, Jianxiang Zhou, Zhiwu Jiang, Kangsheng Li

**Affiliations:** 1 Department of Microbiology and Immunology, Key Immunopathology Laboratory of Guangdong Province, Shantou University Medical College, Shantou, 515041, China; 2 Department of Veterinary Medicine, University of Maryland, College Park, 20742, MD, USA

## Abstract

**Background:**

Phosphatidylinositol 3-kinase (PI3K)/Akt signaling pathway, activated during influenza A virus infection, can promote viral replication via multiple mechanisms. Direct binding of NS1 protein to p85β subunit of PI3K is required for activation of PI3K/Akt signaling. Binding and subsequent activation of PI3K is believed to be a conserved character of influenza A virus NS1 protein. Sequence variation of NS1 proteins in different influenza A viruses led us to investigate possible deviation from the conservativeness.

**Results:**

In the present study, NS1 proteins from four different influenza A virus subtypes/strains were tested for their ability to bind p85β subunit of PI3K and to activate PI3K/Akt. All NS1 proteins efficiently bound to p85β and activated PI3K/Akt, with the exception of NS1 protein from an H5N1 virus (A/Chicken/Guangdong/1/05, abbreviated as GD05), which bound to p85β but failed to activate PI3K/Akt, implying that as-yet-unidentified domain(s) in NS1 may alternatively mediate the activation of PI3K. Moreover, PI3K inhibitor, LY294002, did not suppress but significantly increased the replication of GD05 virus.

**Conclusions:**

Our study indicates that activation of PI3K/Akt by NS1 protein is not highly conserved among influenza A viruses and inhibition of the PI3K/Akt pathway as an anti-influenza strategy may not work for all influenza A viruses.

## Background

Influenza A virus continues to pose a severe threat to poultry farming and human health around the world. To ensure efficient replication in host cells, influenza virus manipulates cellular proteins or hijacks important signaling pathways, of which the PI3K/Akt pathway has received most attention [1,2]. A variety of influenza A virus strains can activate PI3K/Akt signaling pathway to support their multiplication [3-6], which is significantly suppressed by specific PI3K/Akt inhibitors [6-8]. Therefore, targeting the PI3K/Akt signaling pathway is seen as an attractive and promising anti-influenza strategy [9].

Activation of PI3K/Akt during influenza A virus infection can be mediated by diverse mechanisms, such as the interactions between NS1 proteins of some avian influenza A viruses and cellular proteins Crk/CrkL [10]; direct binding and activation of Akt by NS1 [11]; as well as the accumulated viral RNA during the infectious process [12,13]. However, the most important mechanism responsible for the activation of PI3K/Akt signaling is the association between NS1 protein and p85β subunit of PI3K [3-5,14-17]. In the absence of other viral proteins, exogenous expression of NS1 derived from different influenza A virus strains in cells is enough to induce Akt phosphorylation and activation [3,7,10,16]. In contrast to influenza A virus NS1 protein (A/NS1), influenza B virus NS1 protein (B/NS1), which shares less than 20% identity to A/NS1 in amino acid sequence, naturally lacks the potential to induce PI3K/Akt signaling [7].

NS1 does not exist in virus particles, but it is greatly expressed in influenza virus-infected cells, especially in the late phase of infection. The average length of NS1 from most influenza A viruses is 230 aa, however, the length can vary from 202 to 237 aa due to the deletion, truncation, or addition of amino acids. Besides, amino acid substitution is also a common event for NS1 protein, reflecting the evolutionary needs or adaptation of influenza viruses in different species. Several amino acid mutations have been shown to alter NS1 function. For instance, NS1 from different influenza A viruses displayed differential binding to CPSF30 (cleavage and polyadenylation specificity factor 30 kDa) because of the residues replacement at positions 103, 106, 108, 125 or 189 [18-20]. Likewise, sequence variation in NS1 may have variable effects in activating the PI3K/Akt signaling pathway. To address this hypothesis, four NS1 proteins from different influenza A virus subtypes/strains were selected and subjected to a series of comparative analyses. Our data showed that NS1 protein from an H5N1 virus is unable to activate PI3K/Akt, although it can interact efficiently with p85β. Additionally, this H5N1 virus exhibited an enhanced replication upon PI3K inhibition, highlighting an inner correlation between NS1 variation, PI3K/Akt pathway and virus pathogenicity.

## Methods

### Cell lines, viruses, and reagents

Madin-Darby canine kidney (MDCK) cells, human lung carcinoma cell line (A549), and human cervix epithelial cells (Hela) were routinely cultured in Dulbecco’s modified Eagle’s medium (DMEM) supplemented with antibiotics and 10% fetal calf serum at 37°C in 5% CO_2_.

Influenza A virus strains A/Shantou/169/06(H1N1), A/Shantou/602/06(H3N2), and A/Chicken/Guangdong/1/05(H5N1) were used in this study. Viral RNA from A/Quail/Hong Kong/G1/97 (H9N2) virus was kept in our lab. The viruses above are abbreviated hereafter to ST169, ST602, GD05, and Qa97.

QIAamp viral RNA mini kit was purchased from Qiagen (Hilden, Germany); Trizol reagent and Lipofectamine 2000 from Invitrogen (Carlsbad, CA, USA); AMV reverse transcriptase, PrimeSTAR HS DNA polymerase, restriction endonucleases, and T4 DNA ligase from TaKaRa (Dalian, China); plasmid extraction kit and DNA gel purification kit from Tiangen (Beijing, China); and plasmid pGST-p85β expressing human p85β from FulenGen (Guangzhou, China).

Mouse anti-β-actin antibody, rabbit anti-Flag antibody, and peroxidase-conjugated goat anti-mouse antibody were from Sigma (St. Louis, MO, USA); rabbit anti-phospho-Akt(Ser473) antibody and rabbit anti-Akt antibody from Cell Signaling (Danvers, MA, USA); mouse anti-NS1 antibody and mouse anti-p85β antibody from Santa Cruz (CA, USA); peroxidase-conjugated goat anti-rabbit antibody and PI3K inhibitor LY294002 from Beyotime Biotechnology (Jiangsu, China). Mouse anti-NP antibody was produced in our laboratory.

Yeast MATCHMAKER GAL4 two-hybrid system 3 and X-α-gal were purchased from Clontech (Palo Alto, CA, USA); TNT T7 Quick Coupled Transcription/Translation Systems and Transcend Chemiluminescent Non-Radioactive Translation Detection Systems were purchased from Promega (Madison, WI, USA); Protein A/G magnetic beads from New England Biolabs (NEB, Ipswich, MA, USA); protease inhibitor cocktail from Merck (KGaA, Germany); protein G-HRP from Genescript (Piscataway, NJ, USA), and West dura enhanced chemiluminescence reagents from Pierce (Rockford, IL, USA).

### Plasmid construction and confirmation

Full-length NS1 genes from different Influenza A viruses (A/Shantou/169/2006(H1N1), GeneBank: HQ849876; A/Shantou/602/2006(H3N2), GeneBank: HQ849877; A/chicken/Guangdong/1/2005(H5N1), GeneBank: EU874904; A/Quail/Hong Kong/G1/97(H9N2), GeneBank: AF156477) were amplified by reverse transcription PCR using viral RNA and the following primer sets: NS11-S1: 5΄-AATGGATCCATGGATTCCCACACTGT-3΄ and NS11-A1: 5΄-TCGGGATCCTCAAACTTCTGACCTAAT-3΄ for A/Shantou/169/06(H1N1); NS32-S1: 5΄-TA TGGATCCATGGATTCCAACACTGTG-3΄ and NS32-A1: 5΄-TACGGATCCTCAAACTTTTGA CCTAGC-3΄ for A/Shantou/602/06(H3N2); NS51-S1: 5΄-TATGGATCCATGGATTCCAACACT GTG-3΄ and NS51-A1: 5΄-GACGGATCCTCAAACTTTTGACTCAATTG-3΄ for A/Chicken/Guangdong/1/05 (H5N1); and NS92-S1: 5΄-TATGGATCCATGGATTCCAACACTGTG-3΄ and NS92-A1: 5΄-AGTGGATCCTCAAACTTCTGGCTCAAT-3΄ for A/Quail/Hong Kong/G1/97 (H9N2). PCR products were digested with *Bam*HI and inserted into PNF vector (a modified pcDNA3 vector with N-terminal Flag tag) or pcDNA3 vector, giving rise to recombinant plasmids PNF-NS11, PNF-NS32, PNF-NS51, PNF-NS92, pcDNA3-NS11, pcDNA3-NS32, pcDNA3-NS51, and pcDNA3-NS92, respectively. NS11, NS32, NS51, and NS92 are the abbreviations of NS1 protein from H1N1, H3N2, H5N1, and H9N2 viruses. A plasmid PNF-NS51(I) containing 5 aa insert downstream of position 79, was constructed as described previously [21].

To construct NS1-expressing plasmids used for yeast trap assays, the following primers were designed: NS11-S2: 5΄-ACTGAATTCATGGATTCCCACACTGTG-3΄; NS32-S2: 5΄-CGTGAATT CATGGATTCCAACACTGTG-3΄; NS51-S2: 5΄-TATGGATCCTTATGGATTCCAACACTGTG- 3΄; and NS92-S2: 5΄-CGTGAATTCATGGATTCCAACACTGTG-3΄. The reverse transcription PCR reactions were performed using primer sets NS11-S2 and NS11-A1 for A/Shantou/169/06(H1N1), NS32-S2 and NS32-A1 for A/Shantou/602/06(H3N2), NS51-S2 and NS51-A1 for A/Chicken/Guangdong/1/05(H5N1), NS92-S2 and NS92-A1 for A/Quail/Hong Kong/G1/97(H9N2). PCR products were digested with appropriate enzymes and cloned into pGADT7 or pGBKT7 vector to yield plasmids pGAD-NS11, pGAD-NS32, pGAD-NS51, and pGAD-NS92, respectively.

To generate plasmid pGBK-p85β, the full-length coding sequence of human p85β was amplified from plasmid pGST-p85β using primer sets p85β-S: 5΄-GATGAATTCATGGCGGGCCCTGAGG GC-3΄ and p85β-A: 5΄-TTAGAATTCTCAGCGGGCGGCAGGCGG-3΄ by PCR and fused into pGBKT7 vector. All of the constructs were verified by sequencing.

### Western blotting

Cells were lysed with 2 × Laemmli sample buffer (containing 5 mM NaF) in boiling water for 5 min. After brief sonication, the lysates were subjected to SDS-PAGE in 10% polyacrylamide gels and separated proteins were transferred onto nitrocellulose membranes. Membranes were then blocked for 1 h in TBST containing 5% nonfat milk and incubated for 4 h at room temperature with the indicated antibodies. After extensive washes with TBST, membranes were exposed to peroxidase-conjugated secondary antibody (1:3000) for 2 h. Immunoreactive proteins were visualized using West dura ECL reagent and autoradiography.

### Yeast trap assays

Yeast trap assays were performed using the MATCHMAKER GAL4 two-hybrid system 3 according to the manufacturer’s instructions. Briefly, AH109 yeast was transformed with plasmids pGAD-NS11, pGAD-NS32, pGAD-NS51, and pGAD-NS92 along with pGBK-p85β and plated onto SD/-Leu/-Trp media (DDO). AH109 yeasts transformed with plasmids pGBKT7-lam plus pGADT7-T or pGBKT7-P53 plus pGADT7-T were used as negative and positive control, respectively. The plates were incubated at 30°C for about 4 days. Fresh AH109 colonies growing on DDO agar plates were picked and transferred to SD/-Ade/-His/-Leu/-Trp media containing X-α-gal (QDO/X-α-gal) followed by incubation at 30°C for 2 or 3 days. The growth and color of colonies was observed daily. Meanwhile, single AH109 colonies growing on DDO agar plates were put into liquid DDO media and cultured at 30°C overnight (~16-18 h) with shaking (250 rpm). The supernatants were gathered via centrifugation at 14 000 *g* for 2 min and subjected to α-galactosidase activity analysis according to the manufacturer’s protocol.

### GST pull-down analysis

*Escherichia coli* BL21 transformed with pGEX-5x-1 or pGST-p85β plasmid was grown to mid-log phase and induced with 0.1 mM IPTG (isopropyl-β-D-thiogalactopyranoside) at 25°C for 4 h. Bacterial pellets were frozen and thawed for 2 times and lysed with MagneGST lysis reagent containing DNase, lyticase, and protease inhibitors for 40 min. After centrifugation at 14 000 *g* for 10 min, the supernatants were incubated with pre-equilibrated MagneGST beads at 4°C for 30 min. The beads were then washed 3 times with binding/wash buffer and the bound GST or GST-p85β was detected by SDS-PAGE and Coomassie Blue staining.

In vitro translation of different NS1 proteins was performed using pcDNA3-based NS1-expressing plasmids and TNT T7 Quick Coupled Transcription/Translation Systems, according to the manufacturer’s instructions. The translated proteins containing biotinylated lysine residues in their amino-acid sequence were verified by Western blot using Streptavidin-HRP.

Next, GST or GST-p85β beads were incubated with biotinylated NS1 proteins for 2 h at room temperature. After six washes with binding/wash buffer, bound proteins were resolved by SDS-PAGE, followed by Western blot analysis with anti-NS1 antibody.

### Co-immunoprecipitation

Hela cells were transfected with PNF-NS51 plasmid or PNF empty vector. At 36 h post transfection, cells were lysed for 20 min on ice in cold NP-40 lysis buffer (50 mM Tris–HCl, pH 8.0, 150 mM NaCl, 1% NP-40, and protease inhibitor mixture) and centrifuged at 14 000 *g* for 10 min. Supernatant was precleared by protein A/G magnetic beads for 1 h. Then, the sample was mixed with rabbit anti-Flag antibody (1:300) for 2 h at 4°C with rotation. Normal rabbit IgG was used as a control. Protein A/G magnetic beads were added to the mixture and incubated overnight at 4°C with gentle rotation. The beads were washed 3 times with NP-40 lysis buffer, followed by elution of bound proteins with 2 × Laemmli sample buffer in boiling water for 5 min. Western blot analysis was conducted using mouse anti-p85β antibody (1:750) or mouse anti-NS1 antibody (1:1000) and Protein G-HRP (1:2500). Immunoblots were developed using West Dura ECL detection reagents.

### Viral growth kinetics assays

Serum-starved MDCK cells were pre-treated with 20 μM LY294002 for 2 h. Different influenza A virus strains were subsequently added at an MOI (multiplicity of infection) of 0.001. After 1 h of adhesion, the medium was replaced with serum-free DMEM containing TPCK-trypsin and 20 μM LY294002. Supernatants from infected cells were collected at various post-infection time points and titrated by plaque assay in MDCK cells. The experiments were repeated independently 3 times.

## Results

### Differential effects of different NS1 proteins on Akt phosphorylation

**Figure 1:**
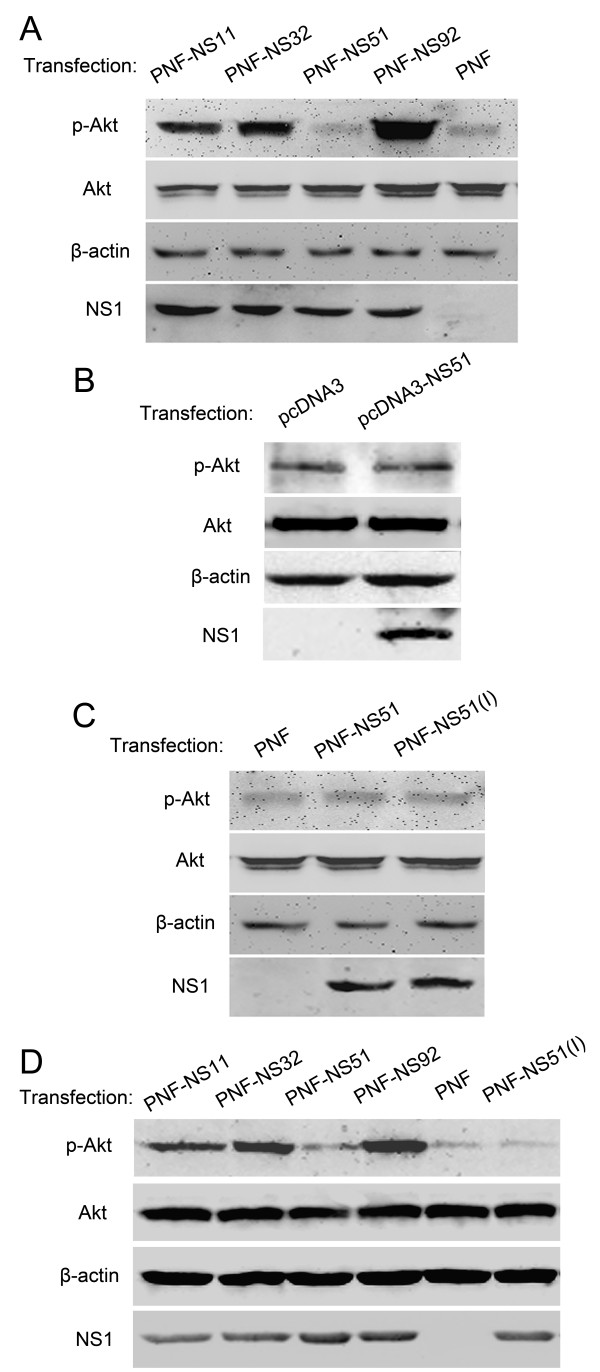
**Effects of exogenous NS1 proteins on Akt phosphorylation in different cells.** Hela (**A**, **B**, **C**) or A549 cells (**D**) were serum-starved for 12 h and transfected with different NS1-expressing plasmids or PNF empty vector. At 24-h post transfection, cells were lysed and subjected to Western blot analysis using the specific antibodies for phospho-Akt(Ser473), total-Akt, β-actin, or NS1.

Activation of PI3K/Akt pathway has been observed upon the transfection of plasmids encoding NS1 proteins from a wide range of influenza A viruses [3,7,10,16]. Moreover, Akt phosphorylation at serine 473 has been reported to be PI3K-dependent [22,23]. Therefore we determined the phosphorylation status of Akt induced by exogenous NS1 proteins from virus strains ST169(H1N1), ST602(H3N2), Qa97(H9N2), and GD05(H5N1). All NS1 proteins except NS51 dramatically enhanced phosphorylation of Akt in Hela cells in our tests (Figure 1a). Especially, NS92 gave rise to the highest level of Akt phosphorylation, whereas NS11 and NS32 induced phosphorylation to a lesser degree. To our surprise, NS51 had no effect on the phosphorylation level of Akt at Ser473 (Figure 1a). This phenomenon has not been reported so far for influenza A virus, except for NS1 from influenza B virus [7].

To rule out the possible influence of Flag tag on NS1 function, pcDNA3-based NS51-expressing plasmid (without Flag tag) was transfected into Hela cells and analyzed by Western blotting. The level of phospho-Akt(Ser473) was the same in both the cells transfected with pcDNA3-NS51 plasmid and the cells transfected with pcDNA3 empty vector, being consistent with the above findings, (Figure 1b).

**Figure 2:**
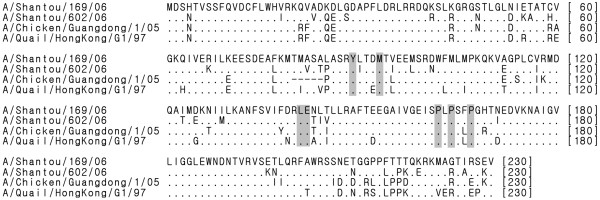
**Sequence alignments of different NS1 proteins.** Amino acid sequence alignments of NS1 proteins from different influenza A virus strains. Numbers correspond to residues in full-length proteins; shadowed boxes denote the key residues (Y89, M93, L141, E142, P162, P164, and P167) responsible for the binding of NS1 to p85β.

Deletion of five residues at positions 80–84 of NS1 is a common phenomenon for most H5N1 viruses isolated after 2000 [24,25]. We also noticed the same deletion in our NS51 protein but not in three other NS1 proteins tested (Figure 2). Thus, we ask whether this deletion could account for the inability of NS51 to activate Akt and we found that NS51 protein with five amino acid inserts still cannot augment the phosphorylation level of Akt at Ser473 (Figure 1c), suggesting that the failure of NS51 to stimulate Akt phosphorylation had nothing to do with this deletion.

To investigate cell type specificity, we further performed phosphorylation assays utilizing the A549 cell (a widely used human alveolar epithelial cell line in NS1 research) and observed similar results (Figure 1d), indicating that the differential effects of different NS1 on Akt phosphorylation were cell type-independent.

### Conservative p85β-binding sites in the NS51 protein

The activation of PI3K/Akt is known to be primarily due to direct interaction between NS1 protein and p85β subunit of PI3K [3-5,14-17]. Since NS51 protein failed to activate PI3K/Akt, a reasonable speculation is that NS51 may not interact with p85β. Therefore, we compared the protein sequence of NS51 to three other NS1 proteins (Figure 2). Strangely, although NS51 displayed some amino acid variation to NS11, NS32 and NS92 (especially in the C-terminus), key amino acid residues, which are reportedly involved in the interaction between NS1 and p85β, such as Y89, M93, L141, E142, P162, P164, and P167 [4,5,16,17], remained unchanged in NS51 (Figure 2).

### Yeast trap assays of the association between different NS1 and p85β

**Figure 3:**
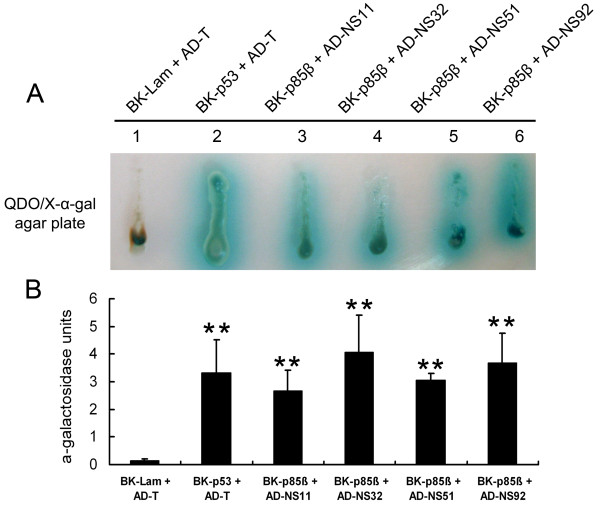
**Analysis of the interactions between NS1 proteins and p85β using yeast trap assays.A**) AH109 yeasts transformed with the indicated plasmids were firstly plated onto DDO plates for 4 days of incubation at 30°C. And then, single AH109 colonies were picked and streaked on QDO/X-α-gal agar plate and incubated at 30°C for 3 days. The representative colonies were presented. (**B**) Different NS1-encoding plasmids were transformed into AH109 yeasts together with pGBK-p85β plasmid for α-galactosidase activities assays in liquid culture using PNP-α-gal as substrate. Data were from three independent experiments. BK-p53 + AD-T and BK-Lam + AD-T represented positive and negative control, respectively. Statistical comparison of different values was performed by the Student *t* test. Groups marked with *asterisks* are statistically different from the negative group (*P* < 0.01).

Yeast trap assays were applied to examine the correlation between NS1 and p85β. For this purpose, four recombinant plasmids (pGAD-NS11, pGAD-NS32, pGAD-NS51, and pGAD-NS92) were transformed into AH109 yeast cells in combination with the pGBK-p85β plasmid. The experimental results showed that, similar to positive control (pGBKT7-p53 plus pGADT7-T, lane 2 in Figure 3a), AH109 yeasts harboring each NS1-encoding plasmid and pGBK-p85β plasmid exhibited vigorous growth and formed characteristic blue colonies in QDO/X-α-gal media (lanes 3–6 in Figure 3a), indicating the activation of reporter gene and the secretion of α-galactosidase in these yeasts. In contrast, AH109 yeasts in negative control (pGBKT7-Lam plus pGADT7-T) did not grow on QDO/X-α-gal media, nor did they produce and secrete α-galactosidase, as shown by the brown dead yeast cells (lane 1 in Figure 3a). Quantitative analysis of α-galactosidase activities in liquid DDO media also proved the comparable level of α-galactosidase generated in experimental groups and positive control (Figure 3b). The above results suggest that, compared to the other NS1 proteins, NS51 did not display obvious variation in its binding capacity to p85β in yeast cells.

### In vitro interaction between NS51 and p85β

**Figure 4:**
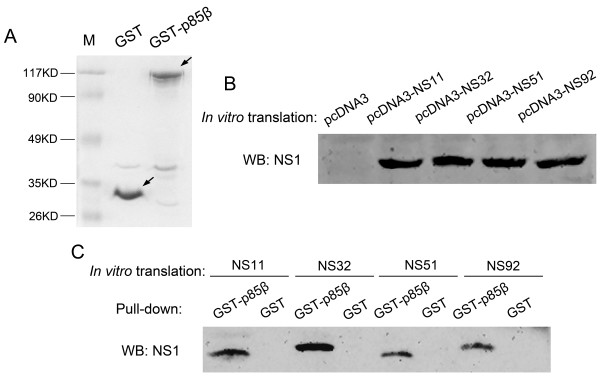
**In vitro binding of NS1 proteins with p85β.** (**A**) Expression and purification of GST and GST-p85β protein in *E.coli* BL21. Purified GST and GST-p85β proteins were resolved in SDS-PAGE and examined by direct staining with Coomassie Blue; M: molecular weight standard. (**B**) In vitro translation of different NS1 proteins using NS1-expressing plasmids and TNT T7 Quick Coupled Transcription/Translation Systems. The in-vitro translated proteins were identified by Western blot using Streptavidin-HRP as the detecting agent. (**C**) GST pull-down assay. GST- or GST-p85β-binding magnetic beads were incubated with NS1 proteins translated in vitro. The bound proteins were subjected to Western blotting with anti-NS1 antibody.

Next, we wanted to investigate whether NS51 directly interacts with p85β in vitro. To this end, GST and GST-p85β were first expressed and purified (Figure 4a), then the four different NS1 proteins were translated in vitro (Figure 4b) followed by the GST pull-down assay (Figure 4c). Our results showed that all four NS1 proteins have been precipitated by GST-p85β instead of GST, giving clear evidence of the association between different NS1 proteins and p85β. Therefore, NS51 protein is capable of binding to p85β in vitro despite its inability to activate PI3K.

### In vivo interaction between NS51 and p85β

**Figure 5:**
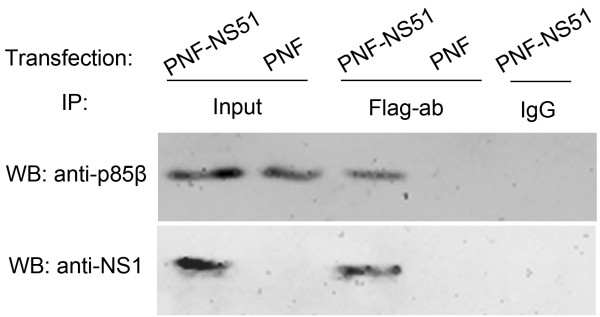
**In vivo association of NS51 with p85β.** Hela cells were transfected for 36 h with either PNF-NS51 plasmid or PNF empty vector. Soluble cellular lysates were immunoprecipitated with rabbit anti-Flag antibody or normal rabbit IgG. Precipitated proteins were separated by SDS-PAGE, followed by Western blot analysis using mouse anti-NS1 antibody or mouse anti-p85β antibody and Protein G-HRP.

To further examine whether interaction between NS51 protein and p85β also exists in vivo, we conducted a Co-IP test and found that Flag antibody precipitated a complex containing Flag-NS51 and p85β from the lysate of Hela cells transfected with PNF-NS51 plasmid (Figure 5), indicating a physical association between endogenous p85β and ectopically expressed NS51 in mammalian cells.

### Dynamics of Akt phosphorylation during infection of different influenza A virus strains

**Figure 6:**
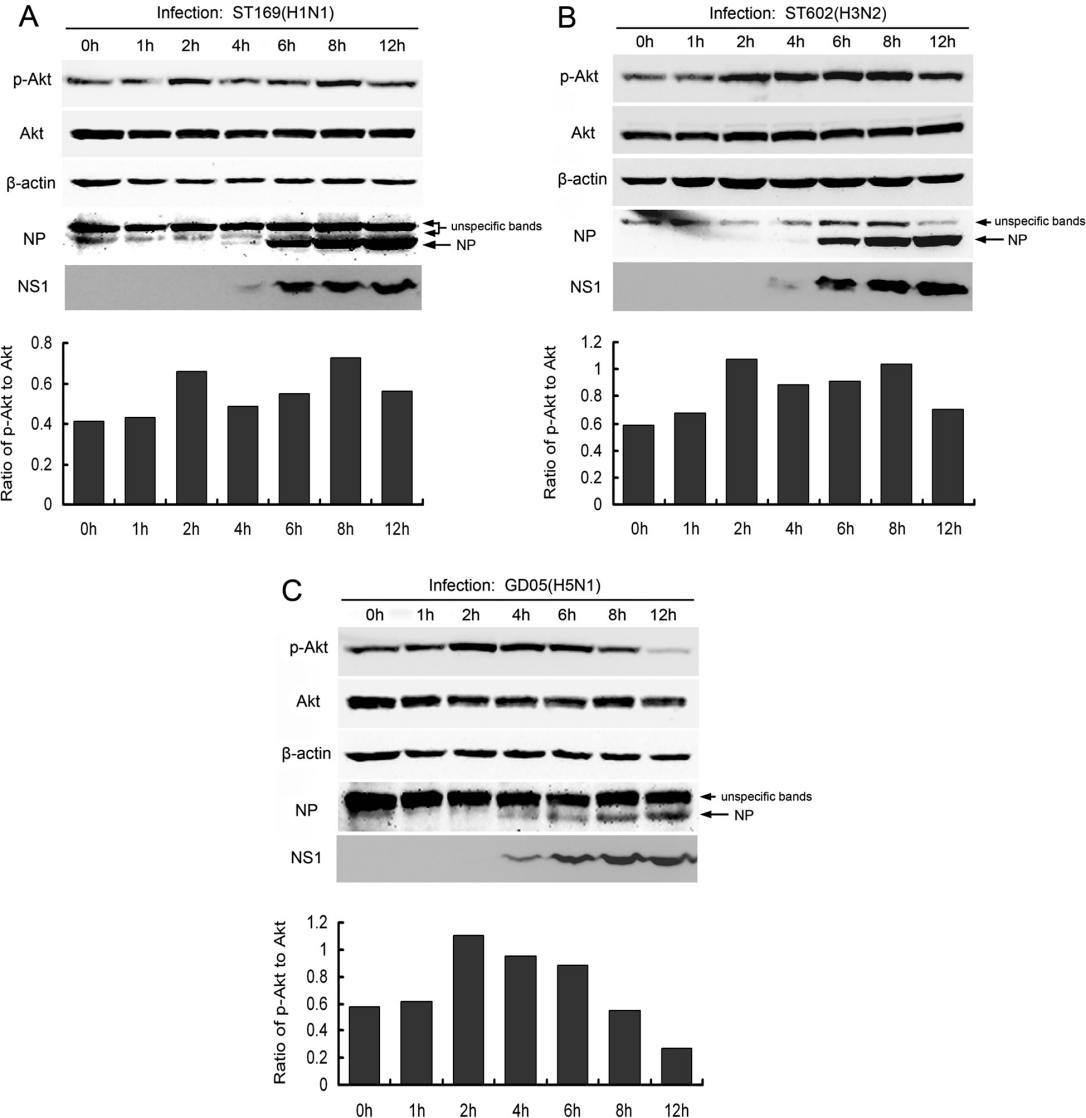
**Variation of Akt phosphorylation during influenza A virus infection.** Serum- starved MDCK cells were infected with different influenza A viruses at an MOI of 2. Cells were lysed at indicated postinfection time points and subjected to Western blotting using specific antibodies for phospho-Akt(Ser473), total-Akt, β-actin, NP, or NS1. Signal intensities of phospho-Akt and total-Akt were quantified by Quantity one software and the ratios were shown at the bottom.

Since NS51 did not activate the PI3K/Akt signaling pathway when exogenously expressed, we further examined its effect on PI3K/Akt in the infection process. We observed that all three tested influenza A viruses upregulated Akt phosphorylation at serine 473 at 2 h postinfection (Figure 6a, b, and c). Additionally, ST169 and ST602 viruses elicited the second elevation of Akt phosphorylation around 8 h postinfection (Figure 6a and b). However, GD05 virus-infected cells did not display increased Akt phosphorylation at 8 h postinfection, albeit NS51 protein was markedly expressed at this time point (Figure 6c). The phosphorylated Akt level even decreased significantly at 12 h postinfection (Figure 6c). We carried out the above experiments twice and got the similar results (data not shown).

### UV-inactivated influenza A viruses induced the early activation of PI3K/Akt pathway

**Figure 7:**
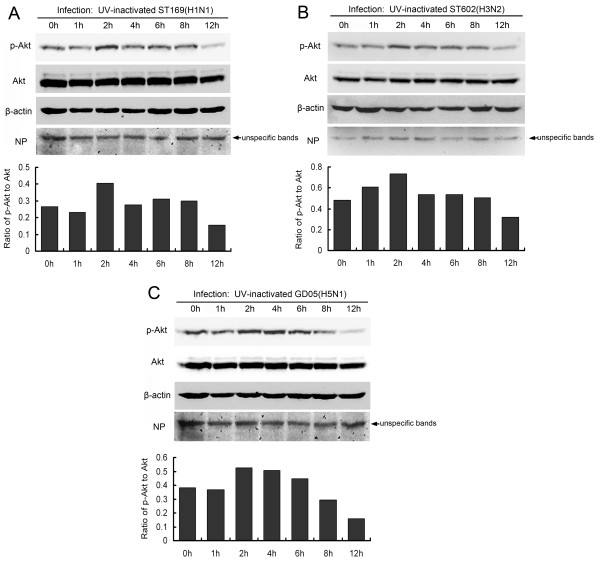
**Akt phosphorylation at early phase of infection induced by UV-inactivated influenza A viruses.** MDCK cells grown in serum-free medium were infected with UV-inactivated influenza A viruses (MOI = 2) for indicated times. Cellular lysates were used for Western blotting using specific antibodies for phospho-Akt(Ser473), total-Akt, β-actin, NP, or NS1. Note that no NP proteins were detected except for unspecific bands. Quantity one software was applied to analyze the signal intensities of phospho-Akt and total-Akt and the relative ratios were presented

The transient activation of PI3K/Akt at the initial stage of infection indicates possible dependence on viral attachment and endocytosis but independent of virus replication. To further clarify this point, influenza viruses were first inactivated by UV irradiation. UV-inactivated viruses can not replicate but still retain the receptor-binding and internalization abilities like wild-type virus [26]. When these inactivated viruses were added into MDCK cell cultures, as expected, they provoked Akt phosphorylation at 2 h postinfection (Figure 7a, b and c), which was maintained for a short time before gradually being reduced with the advancement of infection (Figure 7a, b, and c).

### Suppression of PI3K/Akt pathway imparts different effects on virus replication

**Figure 8:**
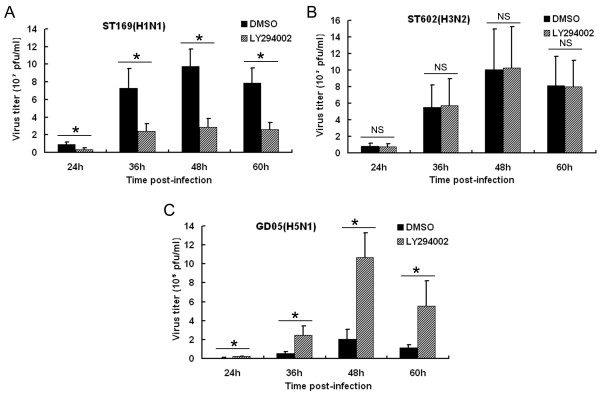
**Diverse effects of LY294002 on the replication of different influenza A viruses.** Serum-starved MDCK cells were treated with LY294002 (20 μM) or DMSO at 2 h before and during infection. Then the cells were infected with different influenza A viruses at an MOI of 0.001. Supernatants were collected at indicated time points and progeny virus titers were subsequently determined by plaque assays and analyzed statistically by one-way ANOVA (*asterisks* indicate *P* < 0.05, NS indicates “not significant”)

Since NS51 was incompetent to activate PI3K/Akt, we presumed that the activation of the PI3K/Akt signaling pathway may be unnecessary for the efficient replication of GD05 virus. To answer this question, a specific PI3K inhibitor, LY294002, was used to examine its suppressive effects on different influenza viruses. ST169 virus exhibited great sensitivity to the treatment of LY294002: relative to the control, virus yield was decreased by a maximum 70% in the presence of LY294002 (Figure 8a). Nevertheless, LY294002 had no obvious influence on the replication of ST602 virus as virus titers in the experimental groups were comparable to those in the control groups at all time points tested (Figure 8b). Most importantly, LY294002 did not suppress but rather substantially promoted the replication of GD05 virus. Virus titer increased up to 5-fold in the LY294002-treated cells compared to the control cells (Figure 8c). Our data suggest the differential effects of PI3K inhibition on the proliferation of different influenza A virus strains.

## Discussion

In the present study, NS11, NS32, and NS92 proteins (expressed either by transfection or infection) efficiently activated PI3K/Akt. However, the NS51 protein did not trigger Akt phosphorylation in both situations (Figures 1 and 6c). Since NS51 had 16.5%, 18.3%, and 12.5% of amino acid sequence diversity relative to NS11, NS32, and NS92 (Figure 2), the failure of NS51 to activate PI3K could be caused by its inability to bind the p85β subunit of PI3K arising from sequence variation. Sequence alignment results show that crucial sites, which have been reported to mediate the interaction of NS1 with p85β (Y89, M93, L141, E142, P162, P164, and P167) [4,5,16,17], are identical among all tested NS1 proteins (Figure 2, shadowed boxes). More importantly, yeast trap assays (Figure 3), GST pull-down (Figure 4) and Co-IP experiments (Figure 5) clearly indicate the interaction of NS51 with p85β. So it can be seen that the binding of NS51 to p85β did not lead to the activation of PI3K/Akt.

PI3K consists of a catalytic subunit (p110) and a regulatory subunit (p85β). In the quiescent state, PI3K remains inactive as the contact between p85β and p110 suppresses the enzymatic activity of p110 [27]. Previous studies reported that interaction of NS1 and p85β forms a heterologous NS1-p85β-p110 trimer [14,15,17], in which NS1 blocks the inhibitory contact between p85β and p110 and leads to the activation of PI3K [15]. Therefore, the binding of NS51 with p85β should activate PI3K. However, this was not the case. NS51 failed to activate PI3K, as shown in our experiments (Figures 1 and 6c). So it is much likely that an as-yet-uncharacterized domain within NS1 could be involved in the activation of PI3K and amino acid mutations in this domain might lead to NS51 binding without activating PI3K. Supporting evidence from Hale’s study is that two amino acid mutations at positions 96 and 97 (E96A/E97A) render loss of PI3K-activating competence of NS1 while retaining its p85β-binding activity [15]. Furthermore, another study has shown that five amino acid mutations (GLEWN to RFPRY) at positions 184–188 entirely deprived the PI3K-activating potential of NS1 [28], which suggests that the 184–188 residues of NS1 are also closely related to the activation of PI3K. But we noticed that E96/E97 (including their flank sequences) as well as 184–188 residues (GLEWN) are considerably conserved in NS51 and three other NS1 proteins (Figure 2). Therefore we believe that there is still another region responsible for PI3K activation. We noticed that 5 residues were missing at positions 80–84 of NS51. Actually, this deletion in the NS1 protein is a popular event for H5N1 viruses isolated after 2000 [24,25]. We then want to know whether it is implicated in the failure of NS51 to activate PI3K/Akt. Our results show that the missed 5 residues in NS51 were not associated with the activation of PI3K/Akt (Figure 1c).

In our study, both wild-type and UV-inactivated influenza A viruses provoked transient Akt phosphorylation at the early phase of infection (Figures 6 and 7), implying that attachment/endocytosis of influenza virus is sufficient for the activation of PI3K/Akt. Similar results regarding the early activation of PI3K/Akt by wild-type influenza A or B viruses have also been reported by others [7,8]. However, it is noteworthy that two independent studies by Shin et al. [6] and Hale et al. [4] showed that UV-inactivated influenza A virus did not induce Akt phosphorylation. The reasons for this discrepancy might be that they examined phospho-Akt at the later time points (6 h postinfection in Shin’s study and 20 h postinfection in Hale’s study) or they used lower MOI (MOI = 1 in Shin’s study) than we did (MOI = 2). Moreover, different influenza virus strains or cell types may contribute to the discrepancy.

Several studies have reported that inhibition of the PI3K/Akt signaling pathway can significantly suppress the replication of influenza A viruses [6-8]. Nevertheless, as was shown in our experiments (Figure 8), different influenza A virus subtypes/strains differed markedly in their susceptibility to the treatment of PI3K inhibitor LY294002. Although 20 μM LY294002 repressed the replication of ST169 virus to a great extent (Figure 8a), it had no apparent effect on the replication of ST602 virus (Figure 8b) and even exerted the opposite effect on GD05 virus (viral titers increased remarkably upon LY294002 treatment, as seen in Figure 8c). Similar to our results, Ehrhardt et al. found that, to efficiently suppress the replication of A/FPV/Bratislava/79(H7N7), the working concentration of PI3K inhibitor was much higher than that required for PR8 suppression (approximately 10–20 folds higher) [8], suggesting that the influence of PI3K/Akt pathway on influenza virus is strain-specific and PI3K activation by NS1 is not of equal importance for the efficient replication of different influenza A virus strains. We did not examine the effects of higher concentrations of LY294002 on influenza A viruses as they exhibited obvious cytotoxicity in MDCK cells (data not shown).

The reasons for the aforementioned phenomena may lie in the dual characters of PI3K/Akt activation. On the one hand, activation of PI3K/Akt signaling pathway benefits influenza virus replication via multiple diverse mechanisms, including preventing cellular apoptosis [3,5,29], promoting viral entry [8], enhancing viral RNA/protein synthesis or favoring nuclear export of viral RNP [6]. On the other hand, the anti-viral function of the PI3K/Akt signaling pathway has also been unraveled by some studies [30,31]. This is probably because PI3K/Akt can mediate the signal transduction of native immunity [8,13,32-35] or enhance expression of anti-viral factors [36]. Accordingly, we speculate that PI3K inhibitor LY294002 may simultaneously induce two opposite effects (pro- and anti-viral effects) during influenza virus infection. Its influence on different influenza viruses is thus determined by which effect is dominant. For the ST169 virus, an inhibitory effect of LY294002 held the prevailing position, so viral replication was markedly suppressed. For ST602 virus, pro- and anti-viral effects of LY294002 were in balance, thus viral replication was not apparently affected. As for GD05 virus, although it stimulated the early activation of PI3K/Akt, it did not induce the late PI3K/Akt activation because of the incompetence of NS51. This hints the late PI3K/Akt activation may not be absolutely essential for the replication of this virus. Therefore, despite LY294002 treatment, GD05 virus titers were increased due to the inhibition of immune response.

## Conclusions

Taken together, activation of the PI3K/Akt signaling pathway is not a conserved property of influenza A virus NS1 protein and inhibition of PI3K/Akt is not always favorable for repression of viral production. Any therapeutic measure targeting a virus-induced signaling cascade (such as the PI3K/Akt pathway) can result in variable antiviral effects due to the numerous subtypes and very high mutation rate of influenza A virus.

## Abbreviations

A549 = Human lung carcinoma cell; Aa = Amino acid; CPSF30 = Cleavage and polyadenylation specificity factor 30kd; DDO = SD/-Leu/-Trp medium; DMEM = Dulbecco’s modified Eagle’s medium; ECL = Enhanced chemiluminescence; GST = Glutathione transferase; Hela = Human cervix epithelial cell; HRP = Horseradish peroxidase; IPTG = Isopropyl-β-D- thiogalactopyranoside; MDCK cell = Madin-Darby canine kidney cell; MOI = Multiplicity of infection; NaF = Sodium fluoride; NS1 = Non-structural protein 1; PI3K = Phosphatidylinositol 3-kinase; p85β = Regulatory subunit of PI3K; p110 = Catalytic subunit of PI3K; QDO = SD/-Ade/-His/-Leu/-Trp medium; RNP = Ribonucleoprotein; SDS-PAGE = Sodium dodecyl sulfate polyacrylamide gel electrophoresis; TBST = Tris Buffered Saline with Tween; TPCK = Tosylamido-2-Phenylethyl Chloromethyl Ketone.

## Competing interests

The authors declare that they have no competing interests.

## Authors’ contributions

WL carried out the GST pull-down, Co-immunoprecipitation experiments and drafted the manuscript. KL designed the experiments and revised the manuscript. GW and HZ performed Western blot experiments. YS participated in the plasmids construction. JD and LW performed yeast trap analysis and viral titer determination. JZ and ZJ assisted in sample preparation. All authors read and approved the final manuscript.
